# The construct validity and reliability of the Turkish version of Spreitzer's psychological empowerment scale

**DOI:** 10.1186/1471-2458-10-117

**Published:** 2010-03-09

**Authors:** Sarp Uner, Sevgi Turan

**Affiliations:** 1 Department of Public Health Hacettepe University Faculty of Medicine, Sihhiye, Ankara, 06100, Turkey; 2 Department of Medical Education and Informatics, Hacettepe University Faculty of Medicine, Sihhiye, Ankara, 06100, Turkey

## Abstract

**Background:**

Today, many organizations have adopted some kind of empowerment initiative for at least part of their workforce. Over the last two decades, two complementary perspectives on empowerment at work have emerged: structural and psychological empowerment. Psychological empowerment is a motivational construct manifested in four cognitions: meaning, competence, self-determination and impact. The aim of this article is to examine the construct validity and reliability of the Turkish translation of Spreitzer's psychological empowerment scale in a culturally diverse environment.

**Methods:**

The scale contains four dimensions over 12 statements. Data were gathered from 260 nurses and 161 physicians. The dimensionality of the scale was evaluated by exploratory factor analyses. To investigate the multidimensional nature of the empowerment construct and the validity of the scale, first- and second-order confirmatory factor analysis was conducted. Furthermore, Cronbach alpha coefficients were assessed to investigate reliability.

**Results:**

Exploratory factor analyses revealed that four factors in both solutions. The first- and second-order factor analysis indicated an acceptable fit between the data and the theoretical model for nurses and physicians. Cronbach alpha coefficients varied between 0.81-0.94 for both groups, which may be considered satisfactory.

**Conclusions:**

The analyses indicated that the psychometric properties of the Turkish version of the scale can be considered satisfactory.

## Background

Empowerment may be a social, cultural, psychological or political process through which individuals and social groups are able to express their needs, present their concerns, devise strategies for involvement in decision-making, and achieve political, social and cultural action to meet those needs [1]. Empowerment is described as power, control, ability, competence, self-efficacy, autonomy, knowledge, development, self-determination and strengthening of the position of one's own group in society [2-4]. Empowerment aims to mobilize frail and disempowered individuals and groups in order to improve their situation and enable them to take control over their own lives [4].

Today, more than 70 percent of organizations have adopted some kind of empowerment initiative for at least part of their workforce. To be successful in today's global business environment, companies need the knowledge, ideas, energy, and creativity of every employee, from front-line workers to the top-level managers in the executive suite. The best organizations accomplish this by empowering their employees to take initiative without prodding, to serve the collective interests of the company without being micro-managed, and to act like owners of the business [5].

Over the last two decades, two complementary perspectives on empowerment at work have emerged: structural and psychological empowerment. The first is more macro and focuses on the social-structural (or contextual) conditions that enable empowerment in the workplace, while the second is more micro in orientation and focuses on the psychological experience of empowerment at work [5,6]. The two perspectives can be distinguished by a focus on empowering structures, policies, and practices and a focus on perceptions of empowerment, and each perspective plays an important role in the development of a theory of empowerment [6].

The structural view focused on empowering management practices includes the delegation of decision-making from higher to lower organizational levels and increasing access to information and resources for individuals at the lower levels. In this structural view, the rationale is that employees will behave in an empowered way by making the necessary changes at the structural level [7]. The social-structural empowerment is about employee participation through increased delegation of responsibility down throughout the organizational chain of command [5].

More specifically, employees would feel more personal control over how to perform the job; would be more aware of the business and the strategic context in which the job is performed; and would be more accountable for performance outcomes [7]. Spreitzer defined these cognitive-affective responses as psychological empowerment [8].

There is a consistent and strong relationship between empowerment cognitions and employees' job satisfaction and organizational commitment. Results indicate that the more employees feel empowered, the happier they are with their job and the more committed to their organization [7]. Directing and increasing individual performance also increases the organization's performance [9]. One of the ways to provide this is to determine how valuable wage earners find their jobs, and how perfect and autarchic they themselves feel. At this point, researches are charged with important duties.

According to Spreitzer, the lack of methods for measuring psychological empowerment in the context of work is one of major causes of obstruction of researches on empowerment [3]. There might be a need to measure psychological empowerment at several levels: at the individual level as well as in organizations and communities [4]. Measuring empowerment in working life is still in the initial phase. There are some instruments for measuring psychological empowerment. Spreitzer's tool, which was developed to evaluate psychological empowerment in the workplace environment, is one such scale, which Spreitzer prepared through the benefit of previously developed instruments and theoretical information; the four dimensions that are mentioned above are discussed.

Spreitzer tested the reliability and validity of her scale. The Cronbach alpha reliability coefficient for the overall empowerment construct was 0.72 and 0.62 for two different groups. In the study, test-retest reliability was examined and the results showed that the stability level was average. A second-order confirmatory factor analysis (CFA) was conducted and an excellent fit for one group and a modest fit for the other group were obtained. The four factors were significantly correlated with each other in both samples [8].

The instrument has been used successfully in different studies in contexts ranging from nurses to low-wage service workers to manufacturing workers [4,10]. According to the results of Arneson and Ekberg research, Spreitzer's questionnaire has undergone the most comprehensive investigation, including measures of reliability and regression analysis as well as the examination of control variables [4]. Kraimer et al provided substantial support for the convergent and discriminant validity of scores on Spreitzer's multidimensional scale on nurses [10].

Hocwalder and Brucefors assessed the Swedish translation of SPES on nurses [11]. The reliability of the sub-scales was between 0.77-0.90. The dimensionality of the scale was evaluated and the four extracted factors explained nearly 70% of total variance. The construct validity of the scale was evaluated and except for the χ^2 ^measure for the two female groups; the fit measures for all groups indicated an acceptable fit between the data and theoretical model. The analyses indicated that the psychometric properties of the scale can be considered satisfactory [11].

Although researches done regarding empowerment have increased in Turkey in the 2000s, most of them concern the theoretical framework [12]. Some preliminary work has been done, intended to adapt the scale to Turkish. Hancer's research examined the dimensions of the Turkish version of SPES, but the sample group included only 214 undergraduate tourism students instead of employees [13]. In their research, scale dimensions were examined through applying explanatory factor analysis and limited information about the scale's validity and reliability was obtained. At the end of the study, self-determination and impact dimensions merged under the same factor, the scale was found as three-dimensional, and Cronbach alpha was determined as 0.84 [13]. In Col's research, which covers 403 faculties from 13 universities, three dimensions of SPES were achieved by explanatory factor analysis, but meaning and competence items merged in the same dimension in contrast to Hancer's study [9]. Reliability coefficient for under dimensions was determined as above 0.80.

Dimitriades also achieved the same results in his research using principle component analysis [14]. The study group included 154 Greek employed students [14]. It was determined that 60.3% of the total variance was explained in three dimensions and self-determination and impact merged under the same factor [14]. Kraimer et al. suggested that Spreitzer's multidimensional model of psychological empowerment should include a direct relationship between self-determination and impact [10].

Because previous studies examined only factorial structure and there was limited information about validity and reliability, a validity study of the Turkish version of Spreitzer's scale is needed. We thus planned to implement the SPES among primary health care personnel and to obtain information about the construct validity and reliability of the scale in this group. The aim of the present study was to assess the psychometric properties of a Turkish translation of SPES. More specifically, we aimed (I) to perform a statistical description of the scale and study inter- and intra-group differences, (II) to estimate the scale's reliability, (III) to study its dimensionality, and (IV) to evaluate its construct validity.

## Methods

### Participants

A total of 381 subjects voluntarily participated in the present study. The participants were recruited from 38 primary health care centres in a district in Ankara. The overall response rate was 67.5%. In order to evaluate the stability of the psychometric properties of the empowerment scale and to study professional differences, the study sample was divided into the following two groups: (1) nurses (n = 260); and (2) physicians (n = 121). Mean age of the nurse group was 35.2 ± 5.7 years (19-62) and all were women. The mean age of the physicians was higher (39.1 ± 6.8 years (25-56)) and 48 were male. The total employment period was higher in nurses (nurses; 14.4 ± 6.3 years, physicians; 12.8 ± 6.3 years), while total employment period in primary health care units was higher in physicians (nurses; 9.6 ± 6.4 years, physicians; 11.0 ± 6.4 years).

### Measures

Empowerment was assessed using the instrument developed by Spreitzer [8]. Spreitzer's questionnaire has undergone the most comprehensive investigation, including measures of reliability and regression analysis as well as the examination of control variables (gender, age, education and unit size) [4].

Psychological empowerment is a motivational construct manifested in four cognitions: meaning, competence, self-determination and impact. Each dimension adds a unique element to the overall construct of empowerment, and the four dimensions represent different facets of the empowerment construct [3,7,8]. *Meaning *is "the value of a work goal or purpose, judged in relation to an individual's own ideals or standards". If employees' hearts are not in their work- if work activity conflicts with their value systems- then they will not feel empowerment. Meaning involves "a fit between the requirements of a work role and beliefs, values and behaviours". *Competence*, or self efficacy, is an individual's belief in his or her capability to perform task activities skilfully when he or she tries. In another word, it reflects an employee's beliefs that they have what it takes to do their job well. *Self-determination *is "an individual's sense of having choice in initiating and regulating actions", and reflects whether individuals see themselves as the origin of their actions. It's the employee's perception on the autonomy in the initiation and continuation of work behaviours and processes. *Impact *is "the degree to which an individual can influence strategic, administrative or operating outcomes at work", and reflects whether individuals feel as though they are making a difference in their organization. The lack of any single dimension will deflate, through not completely eliminate, the overall degree of perceived empowerment [3].

The subjects were asked to rate themselves on the empowerment scale. Spreitzer's measure, comprising four 3-item sub-scales (total 12 items), taps the empowerment dimensions of meaning, perceived competence, self-determination and impact. The scale was translated into Turkish by Hancer [13]. The response scale was a seven-point Likert scale ranging from 1 (strongly disagree) to 7 (strongly agree). For both the four sub-scales and the total scale, average indexes were formed, meaning that the indexes also ranged between 1 and 7. The higher scores indicate the perception of being more psychologically empowered.

### Procedure

A questionnaire (including the empowerment scale) with written instructions was sent to the work address of the participants by the District Health Administration. The questionnaires were sent to all the health personnel in these centres. The aim of the study was explained in the beginning of the questionnaire and subjects voluntarily participated to the present study. No ethical committee approval was sought because observational studies with voluntary participation of adults, together with informed consent, were exempt from further ethical approval. The data were collected over 30 consecutive days.

### Statistical analysis

1. The inter-group differences and intra-group differences with regard to the scale were studied using t-test, one-way ANOVA (dependent measures) with Bonferroni corrections, and a 2 (groups) × 4 (sub-scales) ANOVA (mixed model) with Bonferroni corrections.

2. The reliability of the scale was estimated using Cronbach alpha coefficients. Reliability coefficients greater than 0.80 are considered very good and greater that 0.90 are considered excellent [15].

3. The dimensionality of the scale was evaluated by exploratory factor analyses, using Kaiser's criterion (eigenvalue >1), estimated by the maximum-likelihood method, and rotated with the varimax method [16]. The analyses were made using the SPSS-15.0 program. Furthermore, the dimensionality of the scale was evaluated by CFA, using the maximum-likelihood method and the following measures to assess the fit between the obtained solution and the postulated model: Root Mean Square Error of Approximation (RMSEA), Goodness of Fit Index, (GFI), Adjusted Goodness of Fit Index (AGFI) and chi-square (χ^2^)/degrees of freedom (df). A small RMSEA value corresponds to a good fit. RMSEA values less than 0.05 were used to indicate good fit of the data to the hypothesized models [17-19]. GFI and AGFI values close to 1 indicate a maximally good fit [15,19]. GFI and AGFI values greater than 0.95 were used to indicate good fit [20]. GFI and AGFI values between 0.90-0.95 [15,18,19] and RMSEA values between 0.05 and 0.08 were also acceptable for the model [17-20]. According to Marsh *et al*., it is also acceptable for the model that GFI value is 0.85 and AGFI value is over 0.80 and RMSEA/Root Mean Square Residual (RMR) value is less than 0.08, even less than 0.10 [21]. A small χ^2 ^value corresponds to a good fit [17]. The χ^2^/df ratios of less than 5 were used to indicate acceptable fit to the data [20] and of less than 3 a good fit to the data [15].

4. The construct validity of the scale was evaluated by second-order factor analyses using the maximum-likelihood method and the four above-described measures to assess the fit between the obtained solution and the postulated model [17,19]. The analyses were carried out using the LISREL 8.7.

## Results

The results of the study, which was aimed to assess the psychometric properties of a Turkish translation of SPES, are presented in this section.

### Descriptive statistics and group differences

Table 1 shows the means and standard deviations for the empowerment scale. In all groups, subjects gave the highest self ratings on the meaning and competence sub-scales. Table 1 also presents means that can be used to investigate between-professions and intra-group differences on the empowerment scale. Between-group comparisons for the four sub-scales revealed significant differences between the two groups in competence (p < 0.01), self-determination (p < 0.01) and impact (p < 0.01) sub-scales.

**Table 1:** Means, standard deviations for the scale and group differences on the scale

Scale	Nurses(n = 260) (mean ± sd)	Physicians (n = 121) (mean ± sd)	t	p
**Meaning**	6.57 ± 0.84	6.39 ± 1.11	-1.61	0.11

**Competence**	6.75 ± 0.59	6.36 ± 0.97	-4.03	<0.01

**Self-determination**	5.24 ± 1.62	5.97 ± 1.12	5.09	<0.01

**Impact**	4.32 ± 2.02	5.28 ± 1.63	4.97	<0.01

**Total scale**	5.72 ± 0.92	5.99 ± 0.89	2.82	<0.01

Within-group comparisons for the four sub-scales indicated the following: a comparison of the ratings on the four sub-scales indicated a significant effect for nurses, [F(3, 777) = 234.79, p < 0.01] and for physicians [F(3, 360) = 33.39, p < 0.01]. Multiple post-hoc tests (Bonferroni) showed significant (p < 0.01) differences between all sub-scales except between sub-scales of meaning and competence in each group.

A 2 (groups) × 4 (sub-scales) ANOVA (mixed model) was performed in order to study the main effects and the interaction effect. The ANOVA revealed significant effect due to the group-factor [F(1, 379) = 7.95, p < 0.01]. The sub-scale effect was also significant [F(3, 1137) = 173.03, p < 0.01]. Multiple post-hoc tests (Bonferroni) indicated significant (p < 0.01) differences between all sub-scales except between sub-scales of meaning and competence. Finally, the ANOVA revealed a significant interaction effect [F(3, 1137) = 27.79, p < 0.01]. For nurses, the highest average self-ratings were on the competence sub-scale, followed by meaning, self-determination, and impact; for physicians, the highest average self-ratings were on the meaning and competence sub-scales, followed by self-determination, and impact.

Table 2 shows the Pearson product-moment correlation coefficients between the four sub-scales. For physicians, the correlation coefficients between the four sub-scales were statistically significant and varied between 0.248 and 0.463. For nurses, the correlation coefficients between the competence and impact sub-scales were not statistically significant; other sub-scales were statistically significant and varied between 0.131 and 0.520. Thus, the four scales correlated moderately with each other for physicians and for nurses, with the exception of the competence and impact sub-scales.

**Table 2:** Pearson product-moment correlation coefficients between the sub-scales

		Nurses	(n = 260)		Physicians	(n = 121)
**Scale**	**Me**	**Co**	**Se**	**Me**	**Co**	**Se**

**Competence**	0.520(*)			0.403(*)		
**Self-determination**	0.298(*)	0.131(**)		0.396(*)	0.463(*)	
**Impact**	0.142(**)	0.087	0.520(*)	0.248(*)	0.417(*)	0.414(*)

Me: Meaning; Co: Competence; Se: Self-determination

* *p *< 0.01 (two--tailed)

** *p *< 0.05 (two-tailed)

### Reliability

The internal consistency coefficients as measured by Cronbach alpha. The four sub-scales (meaning 0.86; competence 0.89; self-determination 0.81; impact 0.93 for nurses and meaning 0.90; competence 0.92; self-determination 0.84; impact 0.94 for physicians) and the total scale (nurse;0.83 and physician;0.88) may be considered very good [15].

### Dimensionality

Table 3 shows the results from exploratory factor analyses. The exploratory factor analysis was performed in order to be able to directly inspect whether or not the factor-loading matrix possessed the so-called simple structure. The KMO (Keiser-Meyer-Olkin) value of scale is 0.80 (defined as meritorious) and Bartlet test value is 2033.421 (p < 0.01). Kaiser's criterion indicated that for both factor-analyses, the solutions with exactly four factors should be retained. The four extracted factors explained 80.86% of the total variance for nurses and 84.49% for physicians. The following difference between the solutions of the two groups emerged. In the nurse group, Factor IV explained most of the total variation followed by Factors II, I and III, while in the physician group, Factor II explained most of the total variation followed by Factors IV, I and III. An inspection of the rotated factor loading matrices revealed that, with only some minor deviations, a simple structure was obtained in both solutions. For the nurses, however, item Se3 also loaded substantially (0.485) on Factor IV (impact). For the physicians, the item Im1 (0.315) and Se2 (0.389) also loaded high on Factor II (competence).

**Table 3:** Exploratory factor analysis (maximum likelihood with varimax rotation) of the empowerment scale for the two groups

	Nurses				Physicians			
	FI	FII	FIII	FIV	FI	FII	FIII	FIV
**Me1**	**0.820**	0.241	0.012	-0.020	**0.886**	0.199	0.165	0.116
**Me2**	**0.833**	0.280	0.150	0.102	**0.882**	0.070	0.174	0.041
**Me3**	**0.866**	0.208	0.197	0.068	**0.876**	0.233	0.100	0.105

**Co1**	0.311	**0.869**	0.015	0.024	0.221	**0.859**	0.172	0.193
**Co2**	0.228	**0.859**	0.067	0.025	0.173	**0.893**	0.140	0.213
**Co3**	0.180	**0.879**	0.049	0.038	0.126	**0.859**	0.203	0.134

**Se1**	0.178	0.052	**0.801**	0.251	0.070	0.167	**0.860**	0.198
**Se2**	0.051	0.101	**0.880**	0.102	0.196	*0.389*	**0.693**	0.127
**Se3**	0.143	-0.052	**0.690**	*0.485*	0.231	0.072	**0.878**	0.176

**Im1**	0.032	0.012	0.225	**0.851**	0.093	*0.315*	0.086	**0.870**
**Im2**	0.014	0.059	0.149	**0.940**	0.094	0.143	0.197	**0.923**
**Im3**	0.071	0.034	0.199	**0.933**	0.083	0.097	0.216	**0.920**

**Eigenvalues**	**2.36**	**2.47**	**2.07**	**2.80**	**2.55**	**2.67**	**2.24**	**2.67**

**% of explained variance**	**19.66**	**20.55**	**17.28**	**23.37**	**21.21**	**22.38**	**18.67**	**22.23**

**Cumulative % of explained variance**				**80.86**				**84.49**

Me: Meaning; Co: Competence; Se: Self-determination; Im: Impact.

Note: Factor loadings for items that define a given factor are written in boldface. Factor loadings of items that do not define a given factor but are above 0.30 are typed in italics.

Table 4 presents the results from the CFA. The CFA was performed to statistically test the structure found in exploratory factor analysis. The χ^2^-value in Table 4 indicates that the factor solution significantly deviates from the postulated four-factor model. This point to the rather well-known drawback of the χ^2 ^test in the factor analytical context. It is usually the case that for large samples, small deviations of the empirical data from the theoretical model lead to a significant deviation [17]. However, χ^2^/df ratio (nurse, 1.87; physician, 2.08) indicated a good fit for both groups. According to the criteria for RMSEA, GFI, and AGFI, the factor solutions for all three groups may be considered acceptable in the nurse group. However, in physicians, the criteria for GFI and AGFI may be considered acceptable, but the RMSEA criterion is at the limit.

**Table 4:** Confirmatory factor analysis (maximum likelihood with correlated factors) of the empowerment scale for the two groups (nurses& physicians).

	Nurse				Physician			
	FI	FII	FIII	FIV	FI	FII	FIII	FIV
**Me1**	0.71				0.92			

**Me2**	0.87				0.81			
**Me3**	0.88				0.87			

**Co1**		0.93				0.90		
**Co2**		0.81				0.92		
**Co3**		0.82				0.84		

**Se1**			0.75				0.81	
**Se2**			0.67				0.73	
**Se3**			0.85				0.88	

**Im1**				0.79				0.86
**Im2**				0.94				0.95
**Im3**				0.96				0.92

**χ^2^**				89.91*				99.70*

**χ^2^/df**				1.87				2.08

**RMSEA**				0.05				0.09

**GFI**				0.95				0.88

**AGFI**				0.91				0.80

* *p *< 0.001

### Construct validity

**Figure 1:**
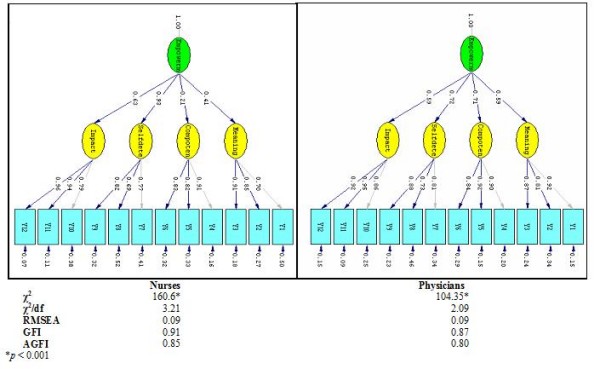
**Results from the second-order factor analysis for the two groups (nurses & physicians)**.

The construct validity of the scale was assessed by second-order factor analyses [17,19]. The purpose of the second-order factor analysis is to study how strongly the first-order factors load on the hypothesized second-order factor, that is, to estimate to what extent the four factors of meaning, competence, self-determination, and impact can be accounted for by the more generic concept of empowerment. Furthermore, the second-order factor analysis can be used to study construct validity because it also assesses the degree to which some items load on a certain hypothesized first-order factor and at the same time load very insignificantly on other first-order factors. Figure 1 presents the results from the second-order factor analyses for both groups. An inspection of the figure reveals that for all groups, the first-order factors (meaning, competence, self-determination, and impact) load significantly on the second-order factor (empowerment). Also, all items load significantly on their hypothesized first-order factor. Except for the RMSEA measure for the two groups, the fit measures for all groups indicate an acceptable fit between the data and the theoretical model. The RMSEA measure for the two groups is at the limit. In sum, these results provide support for the construct validity of the empowerment scale.

## Discussion

The existing study evaluated the psychometric properties of a Turkish adaptation of Spreitzer's empowerment scale [8]. The evaluation was conducted in two different groups: nurses and physicians. One might consider the two parallel analyses as two separate evaluations or applications that jointly outturned the following results.

It is possible to appraise the empowerment scale for both groups as highly reliable, as demonstrated by Cronbach alpha coefficients. As may be recollected, the alpha coefficients with regard to the four sub-scales and the total scale varied between 0.81 and 0.94. These findings are in accordance with both Spreitzer's [8], Kraimer et al.'s [10] and Hocwalder and Brucefors's [11] conclusions.

For all of the groups, the factor solutions brought about exactly four factors that justified over 80 percent of the total variance. In the group of nurses, Factor IV (impact) explicated most of the total variation, while Factor II (competence) explicated most of the variation in the physician group.

For both groups, the scale might be regarded as having construct validity, as indicated by the second-order factor analysis. As Figure 1 points out, all second-order loadings (y) were high (ranging from 0.69 to 0.95) and all first-order loadings (π) were also high and statistically important (varying between 0.21 and 0.93). These results are also compatible with Spreitzer's [8], Kraimer et al.'s [10] and Hocwalder and Brucefors's [11] conclusions.

The comparison of the scores by the two groups in terms of scale dimensions revealed that "Meaning" and "Competence" were high in the nurse group, whilst "Self-determination" and "Impact" were high in the physicians group. It has also been noted that there were statistically considerable differences in all dimension between groups with the exception of "Meaning" dimension. "Competence" is the sole significant unexpected difference between the groups; it would have normally been expected that the physicians are more empowered than the nurses by reason of their longer education, higher salary, greater responsibility and greater authority. This rather unexpected result might be associated with the fact that nurses are more experienced, and perceive themselves as more competent. The decision-making and managerial positions effectuated by the physicians in primary healthcare centers would be taken into account during the interpretation of the differences with regard to "Self-determination" and "Impact".

The nurses have graded themselves highest in terms of competence, and meaning, self-determination and impact respectively. Physicians rated themselves highest with regard to meaning, rather than competence, and the other subgroups yielded similar results to those observed in the nurses. For the nurse group, there were significant (p < 0.01) discrepancies among all sub-scales, while there were significant (p < 0.01) discrepancies among all sub-scales, except those between meaning and competence for physicians. These findings match the results of the previous studies [8,10,11]. Hocwalder and Brucefors described this state as a process, in which the cognitions of meaning and competence are perceived as personal or internally dependent and subjective; and self-determination and impact as work-environment related or externally dependent and objective [11].

The results of the present study were compared with the results of three other studies that are actually the only other examples of assessment of psychometric properties of empowerment scale [8,10,11]. It must however be underlined that there were differences between the backgrounds of the samples covered by the said studies; in terms of culture (Turkey vs. Sweden and USA), language (Turkish vs. Swedish and English) and occupational groups (nurses and physicians vs. insurance and industrial employees). The final results were still similar with respect to the two main psychometric properties of scale, namely reliability and construct validity.

There was, however, a limitation in both the present study and the ones carried out by Kraimer et al. and Hocwalder and Brucefors [10,11]. All of the subjects were primary healthcare personnel (nurses and physicians), and only a few of them were men. In the present study, 87.4 percent of the subjects were women and the female preponderance was also highlighted by Kraimer et al. (90 percent) and Hocwalder and Brucefors (94.6 percent) [10,11].

Another shortcoming of the present study was the emergence of the "ceiling" effect in meaning and competence sub-scales, as in the other studies [8,10,11]. The distortion may be due to the fact that the respondents had relative homogeneity in terms of education and work-environment.

## Conclusions

The present study aims to assess the psychometric properties of a Turkish adaptation of Spreitzer's empowerment scale [8]. In conclusion; the psychometric properties of the Turkish adaptation of SPES, by common standards, can be considered satisfactory. The findings of this study verified and broadened those attained by the other studies that also had the objective of evaluating the psychometric properties of the empowerment scale [8,10,11].

It is important to note that this study aimed to test only the reliability and construct validity of the Spreitzer's scale but not the face, concurrent and convergent validities, which need to be conducted in future studies. The adaptation and evaluation of the scale will hopefully stimulate further research on empowerment in the fields of psychometrics and work life and health psychology in Turkey.

## Competing interests

The authors declare that they have no competing interests.

## Authors' contributions

SU designed and developed the research study, managed the research and edited the manuscript and wrote the manuscript. ST designed and developed the research study, input and analysed data and assisted in writing the manuscript. All authors have read and approved the final manuscript.

## Pre-publication history

The pre-publication history for this paper can be accessed here:

http://www.biomedcentral.com/1471-2458/10/117/prepub
